# Lifelong learning of cognitive styles for physical problem-solving: The effect of embodied experience

**DOI:** 10.3758/s13423-023-02400-4

**Published:** 2023-12-04

**Authors:** Kelsey R. Allen, Kevin A. Smith, Laura-Ashleigh Bird, Joshua B. Tenenbaum, Tamar R. Makin, Dorothy Cowie

**Affiliations:** 1 Department of Brain and Cognitive Sciences, MIT and Center for Brains, Minds, and Machines, Cambridge, MA USA; 2 Department of Psychology, Durham University, Durham, UK; 3 MRC Cognition Brain Sciences Unit, University of Cambridge, Cambridge, UK; 4 Institute of Cognitive Neuroscience, University College London, London, UK

**Keywords:** Cognitive development, Embodied cognition, Motor planning/programming

## Abstract

‘Embodied cognition’ suggests that our bodily experiences broadly shape our cognitive capabilities. We study how embodied experience affects the abstract physical problem-solving styles people use in a virtual task where embodiment does not affect action capabilities. We compare how groups with different embodied experience – 25 children and 35 adults with congenital limb differences versus 45 children and 40 adults born with two hands – perform this task, and find that while there is no difference in overall competence, the groups use different cognitive styles to find solutions. People born with limb differences think more before acting but take fewer attempts to reach solutions. Conversely, development affects the particular actions children use, as well as their persistence with their current strategy. Our findings suggest that while development alters action choices and persistence, differences in embodied experience drive changes in the acquisition of cognitive styles for balancing acting with thinking.

**Supplementary Information:**

The online version contains supplementary material available at 10.3758/s13423-023-02400-4.

## Introduction

Everyday experience is both constrained and enabled by the bodies we inhabit. Taller people can reach further, while people with two fully functioning hands can manipulate multiple objects at the same time. ‘Embodied cognition’ (Wilson, 2002) suggests that such constraints play a fundamental role in shaping our cognitive and perceptual experiences. Many versions of embodiment theory suggest that these effects reach even further, into how we reason about those experiences. Supporting this view, researchers have shown that when individuals’ bodies or skills are altered, e.g., through temporary training or by being born with limb differences, this can change their perceptual capacities (Aglioti et al., 2008; Hagura et al., 2017), spatial cognition (Makin et al., 2010), body representation (Maimon-Mor et al., 2020), or motor skills (Maimon-Mor et al., 2021). Here we ask if these effects of embodiment can be broader, by testing whether differences in embodied experience (through limb differences) affect the ways that people think about acting in the world, even when their capacities for action are made equal.

**Fig. 1:**
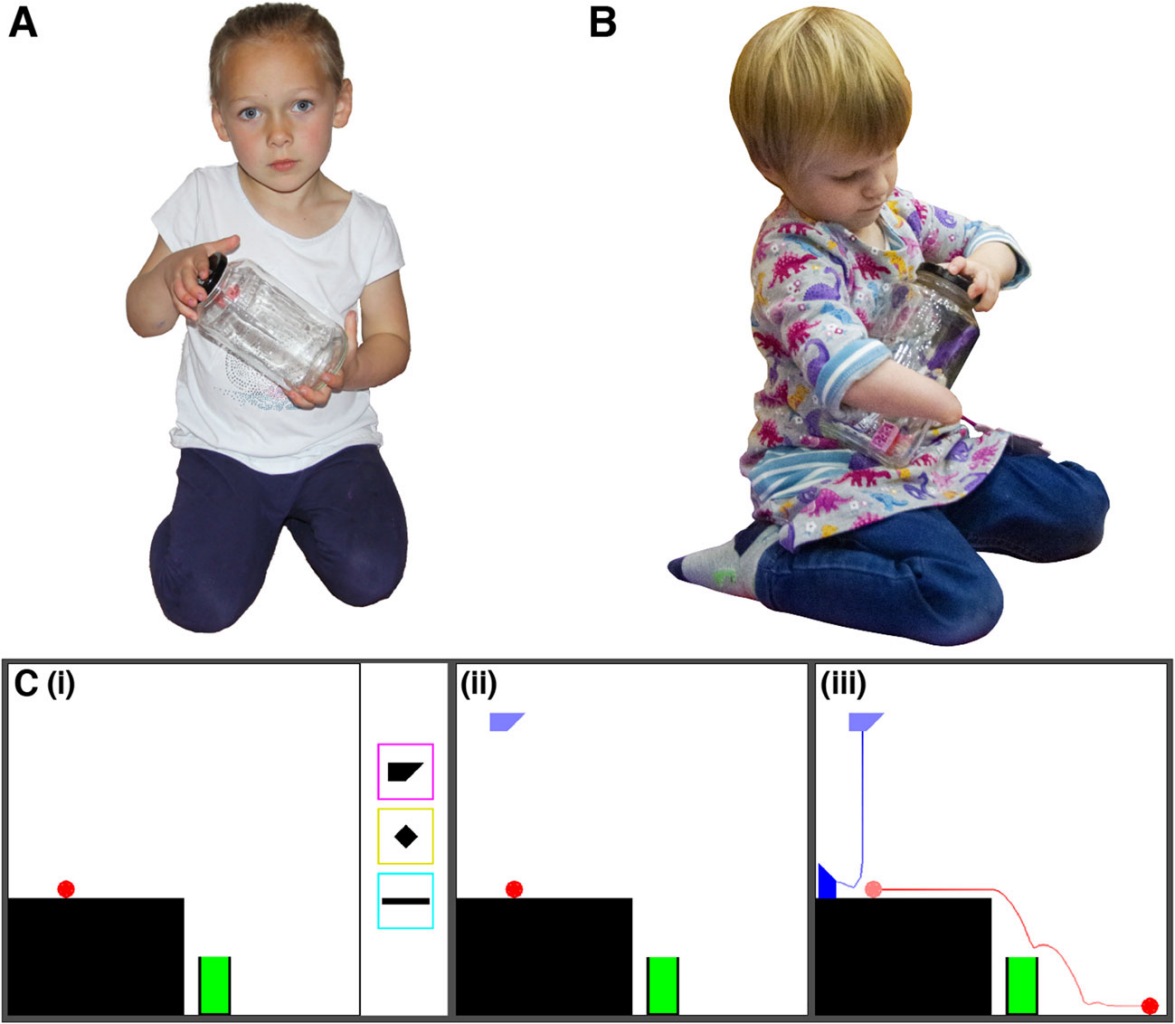
Individuals with limb differences must engage in independent motor problem-solving for many everyday behaviors, such as opening a jar. These experiences may change their cognitive styles for motor tasks in general. For example, with two hands, opening a jar can be accomplished by using one hand to stabilize the jar while the other one twists the lid (**A**). With a single hand (**B**), opening a jar can be accomplished by using one’s arm and torso to stabilize the jar. The Virtual Tools game (**C**) equalizes action possibilities and costs for individuals with different types of limbs by creating a virtual action space. (i) The aim is to move the red ball into the green goal. A participant selects a tool from three options (shapes in colored boxes) and places it in the scene (ii). Once placed, physics is “turned on” and objects can fall under gravity or collide with each other (iii); the blue and red lines represent the observed motion trajectories for the tool and the ball, respectively

Prior studies have rarely addressed how a lifetime of embodied experience affects the use of “cognitive styles”: the individual differences in how people allocate cognitive resources to thinking about and acting in the world (Messick, 1976; Kozhevnikov, 2007). Through short-term direct manipulations of people’s bodies or accessible actions, researchers have shown that people are sensitive to action costs – the amount of effort required to perform actions – for both motor planning (Izawa et al., 2008) and motor behaviors (Prévost et al., 2010). Priming people with different action costs in a perceptual decision-making task can even affect their decisions in a setting where action costs no longer apply (Hagura et al., 2017), suggesting that action costs can generalize beyond the immediate task. However, these studies transpire on the scale of minutes, and typically demonstrate behavioral effects where costs are manipulated within a task but do not show that long-term and generalized action costs are learned from embodied experience.

To study the effect of embodied experience over longer time-scales, researchers have investigated the perceptual and motor capabilities of individuals born with limb differences. However, the tasks used to study these capabilities often require judgments related to absent body parts, and therefore differences in behavior might be driven by differences in sensorimotor experience or available information. For example, while people with congenital limb differences are slower to judge whether a picture is of a left or right hand (Maimon-Mor et al. , 2020; though cf. Vannuscorps and Caramazza , 2016; Vannuscorps et al. , 2012), they lack first-person experience of that hand.

Here we test the hypothesis that growing up with a different body may affect the everyday cognitive styles individuals use to solve problems in their environments, even when the capabilities tested are divorced from particular bodily differences. For example, if people with congenital limb differences have learned that actions are in general more costly – perhaps as a result of difficulties using artifacts designed for people with two hands (see Fig. 1) – we might expect that they will differ in how they approach physical problems generally, even when action costs are equated. Such cognitive styles for action have been observed previously on shorter time-scales: e.g., individuals adapt their motor plans to their own levels of sensorimotor uncertainty and variability (Harris and Wolpert, 1998; Gallivan et al., 2018; Körding and Wolpert, 2004), including becoming more persistent (Leonard et al., 2017), or spending more time thinking before acting (Dasgupta et al., 2018). The differences in cognitive styles might be expected to emerge early in infancy, when experience begins driving motor skill acquisition (Adolph et al., 2018) but might also develop throughout childhood alongside more precise motor planning, control and tool use (Berard et al., 2006; Chicoine et al., 1992; Adalbjornsson et al., 2008).

To test the influence of embodied experience on the learning of cognitive styles, we studied behavior in a *virtual* physical problem-solving task where all participants had equal capabilities to interact with the world. This “Virtual Tools game” (Allen et al., 2020) requires people to use virtual objects as tools to solve a physical problem (e.g., getting the red ball into the green goal, Fig. 1C) using a single limb to control a cursor, thus equating action costs. Allen et al. (2020) provide a set of performance metrics for this task that measure the cognitive styles participants use.

We chose participant groups to represent a diverse range of embodied experience: children (5 to 10-year-olds) and adults born with limb differences, and age-matched children and adults born with two hands. Children have less embodied experience than adults, while individuals with limb differences have dramatically different kinds of experience. By using a virtual task with simple controls, we equate manipulation capabilities and instead study how embodied experience affects the cognitive styles that support action planning and reasoning more generally.

We tested whether cognitive styles are affected by life experience, as indexed by age (children versus adults) and by limb differences. We first predicted that those having to devise unique solutions to everyday physical problems due to growing up with a limb difference might use different, perhaps even more efficient, ways of solving the virtual puzzles. To assess this, we considered the key outcome measures of attempt type, thinking time, attempts to solution, time to solution, and solution rate introduced by Allen et al. (2020). Second, given that tool use capabilities develop throughout childhood (Beck et al., 2011; Keen, 2011), we expected solution rates to improve with age. Finally, we tested whether differences in cognitive styles between those with and without limb differences would emerge early because compensatory behavior evolves early, or whether differences might grow with development as motor and cognitive skills develop. Overall, we found that participants with limb differences do use a different set of cognitive styles than those without – spending more time thinking while interacting less with the world. While performance does improve with age, we did not find evidence that the thinking/acting difference between participants with and without limb differences changes over development.

## Methods

### Participants

We recruited a total of 145 participants across four groups: 40 adults without limb differences (Adult-NLD), 35 adults with limb differences (Adult-LD), 45 children with no limb differences (Child-NLD), and 25 children with limb differences (Child-LD). LD and NLD participants were well matched for age (Child-LD mean: 7.91 years old, sd: 1.84, range 5.08–10.72; Child-NLD mean: 7.94 years old, sd: 1.74, range 5.02–10.56; Adult-LD mean: 40.7 years old, sd: 15.5, range 19–76; Adult-NLD mean: 41.2 years old, sd: 15.2, range 18–70). We extensively liaised with the limb-difference community, including through existing volunteer databases and two UK charities who support children with a limb difference and their families. We included participants with congenital upper limb anomalies, as summarized in Tables S2 and S3 in the Supplemental Material. Information on limb differences was self-reported but verified for a subset of participants (88% and 55% in children and adults, respectively). We recruited children and adults without limb differences to match the education level and age of the special population over the same period. We recruited a larger sample of children without limb differences to give us a greater understanding of the range of typical performance in a two-handed population. Two-handed children were recruited through an existing university volunteer families database and affiliated Facebook page. The experiment was approved for adults under protocols approved by UCL (9937/001), and all adults provided informed consent. The experiment was approved for children under a protocol approved by the ethics committee at Durham University (PSYCH-2019-08-30T10_08_45-mnvj24), and informed consent was provided by the legal guardian.

**Table 1:** Proportion of children and adults with limb differences who have an affected right or left arm, or both arms, and associated ages of groups in years

Age group	Affected limb(s)			Mean (SD) age
	Right arm	Left arm	Both	
Children	0.32	0.44	0.24	7.91 (1.84)
Adults	0.42	0.55	0.03	40.7 (15.5)

### Experiment

As with Allen et al. (2020), the experiment was run online on participants’ personal computers at home. All participants were provided with an identifying code used to link their performance with individual information (e.g., specific limb differences). The experiment progressed through two stages: motor pre-test, and Virtual Tools game. We also collected several additional demographic and clinical details (see below Table 1).

All participants were given the same experiment, with only three exceptions that differed between children and adults: (1) children received simplified instructions for all stages, (2) adults played one additional Virtual Tools level that we removed from the children’s experiment due to excessive challenge (see Section S3 in the Supplemental Material), and (3) adults were given a more extensive questionnaire that included additional questions about the strategies they had used and video games they had played before.

**Fig. 2:**
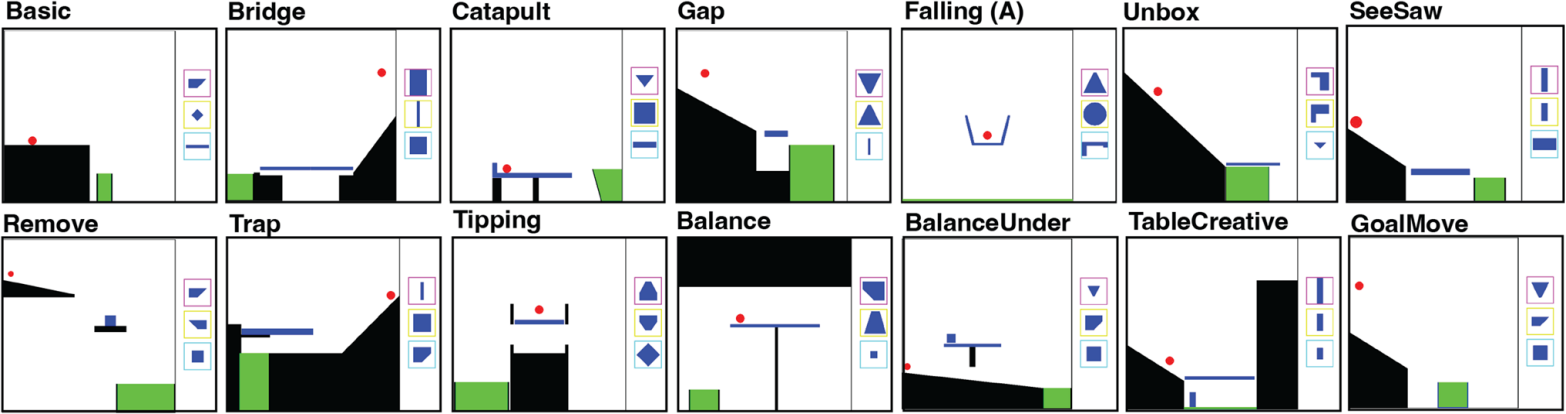
The 14 levels of the Virtual Tools game (Allen et al., 2020) that participants played. These cover a wide variety of physical action concepts including “balancing,” “launching,” “catapulting,” “supporting,” and “tipping.” To play the game, please see https://sites.google.com/view/virtualtoolsgame

#### Motor pre-test

The motor pre-test was used to measure participants’ ability to control the cursor. Each trial began with a star centered in a 600x600 *px* area on the screen. Once the star was clicked, a 10-*px* radius circle appeared in a random position either 150 *px* or 250 *px* from the center. Participants were instructed to click on the circle as quickly and accurately as possible. Participants completed ten motor test trials (five at each distance from the center).

On each trial we measured (a) reaction time, and (b) the distance (in *px*) between the center of the circle and the cursor click location. As a measure of participants’ basic motor accuracy, we took the median of both of those measures across all ten trials; we used the median to avoid skew from outlier trials, and found in pilot testing that this was a relatively stable measurement.

#### Virtual Tools game

On each level of the Virtual Tools game, participants were presented with a scene and three “tools” (see Fig. 1C-i), along with a goal condition (e.g., “get the red object into the green goal area”). Participants could accomplish this goal by clicking on a tool and then an unobstructed part of the game area to place that tool (Fig. 1C-ii). They could choose the location of the tool but its orientation was fixed to how it was displayed on the screen. As soon as the tool was placed, physics was “turned on” and objects could fall under gravity or collide with each other depending on the specific tool placement made (Fig. 1C-iii). If the goal was not accomplished, participants could press a button to reset the scene to its initial state. Participants could attempt to solve the level as many times as they liked but were limited to a single tool placement for each attempt. Participants could move onto the next level once they had accomplished the goal, or after  60 *s* had passed.

Following Allen et al. (2020), participants were initially given instructions about how the game functioned, including the difference between static (black) and moving (blue/red) objects, goal areas, and how to place tools and reset the level. For familiarization with the interface and physics, participants were given one introductory level that required them to place tools at least three times without a goal, followed by two simple levels that they were required to solve that were not analyzed. For adults, this process was identical to that of Allen et al. (2020); children received simplified instructions (see Fig. S1 in the Supplemental Material).

**Fig. 3:**
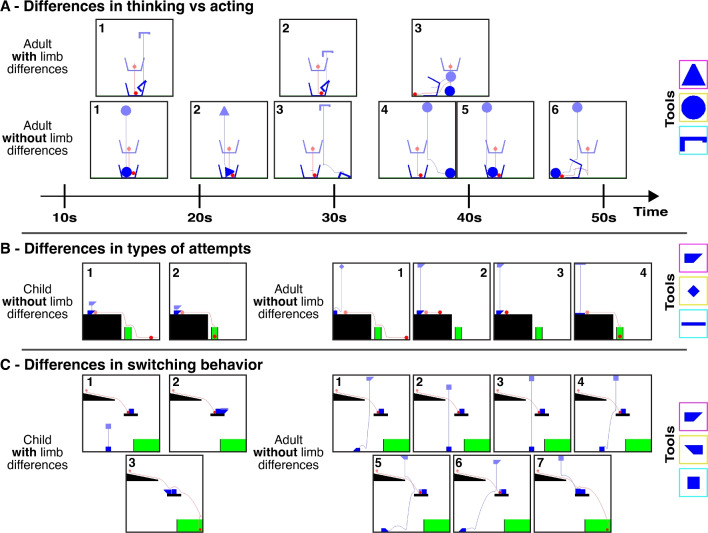
Examples of different participant trajectories through different levels. Each panel is an individual attempt by a participant, labeled by the attempt number. The starting positions of objects are shown as more transparent, while their final positions after physics is “turned on” are shown as opaque. Available tools for the levels depicted are shown on the right. (**A**) Participants with limb differences tend to spend more time between attempts while taking fewer overall attempts to solve levels. In this level, the goal is to knock the container over so that the ball touches the ground. An adult with a limb difference first uses the hook object to try to do this but by the third attempt realizes they can place an object underneath the container to tip it over. An adult without a limb difference persists with placing objects above the container in rapid succession until they are ultimately successful. (**B**) In this level, the goal is to get the red ball into the container. Children often do so by placing a tool very close to the ball, while adults are more likely to drop a tool from further up. Both this child and adult are ultimately successful. (**C**) Adults tend to perseverate with attempt types more than children. In this level, the goal is to get the red ball into the container, which can be achieved by placing a tool on the platform next to the block. Before finding solutions, this child switched between attempt types (from dropping a block at the bottom, to putting a wedge on the wrong side of the platform, to finally putting it on the correct side of the platform) more than the adult (who focused on the same attempt type but tried this attempt type many times before being successful)

In the main task, participants were asked to solve 14 different levels, each with a different set of three tools, designed to probe knowledge of diverse physical principles (e.g., support, collisions, tipping; see Fig. 2). The solutions to each level are determined by the physics of the game, rather than being decided by the experimenters. Solution (“truth”) maps are provided in the supplement (Fig. S2). As in Allen et al. (2020), on each level, we recorded all attempts from each participant – defined as the tool chosen and where it was placed; the time elapsed between the start of the level and the time of the attempt; and whether the level was solved. Examples of participants’ play in different levels is shown in Fig. 3.

For the main analyses, we used four different overall performance metrics defined by Allen et al. (2020) to measure different facets of performance: (1) whether the level was solved (solution rate), (2) time until the solving attempt was performed (time to solution), (3) how many attempts were taken until the level was solved (attempts to solution), (4) times to the first attempt and the average time between attempts (thinking time). We also analyzed the specific kinds of attempts taken (attempt type) using the same methodology introduced in Allen et al. (2020) to cluster and classify participants. All measures were automatically extracted from the recorded tool placements and timings.

#### Additional measures

After both the motor test and Virtual Tools task, participants were given a short questionnaire to ask what device they used to control the cursor, and, for participants with limb differences, how the cursor was controlled. Additionally, for the adult participants we included the questions asked in Allen et al. (2020), including prior video game experience, and free-form responses about strategies they had used on the task. In separate surveys, we gathered demographics and interface information (Section S2.2 in the Supplemental Material), limb-differences information (Section  S2.3 and Tables S2 and S3), and verbal and nonverbal IQ (for a subset of children only; Section S2.1). Age was used as a covariate in our analyses, while gender, device, and limb usage were studied as possible moderators (see Section  S8 in the Supplemental Material). Free-form strategy descriptions were read in order to discover non-standard ways of solving the levels but were not directly analyzed.

Finally, in exploratory analyses of attempt types, we also directly analyze the kinds of errors different groups of participants made in Section  S6. These errors include using an unworkable tool, placing the tool above vs. below the correct object, or placing it at the wrong precise location relative to the correct object.

### Analysis

For the motor test, performance was analyzed using linear models to predict the dependent variable (either median reaction time or click distance) as a function of both age group and limb difference group, controlling for the effect of differences due to age in years separately for adults and children.

For all of the Virtual Tools performance metrics, variables were modeled using linear mixed effect models, using random intercepts for participants and levels. Additionally, we included age in years and median motor test response times as covariates, parameterized separately for children and adults. We had prespecified these as covariates since we believed that they would account for general performance differences; nonetheless analyses without covariates produced similar results (see Section  S7 in the Supplemental Material). For all but the solution metric, we conditioned our analyses only on *successful* levels, as we were interested in the mental processes that led to solutions, and not processes that might be indicative of frustration or perseverance; however, analyzing all levels produced a qualitatively similar pattern of results (see Section S9 in the Supplemental Material).

In some analyses we attempt to differentiate the *type* of participants’ attempts, either to test whether the type of attempt is different across groups, or to test whether participants are switching types between attempts. Measuring attempt type directly in this game is challenging, as different combinations of tools / positions can have overall similar effects (see, e.g., Fig. 3. Which of these should be considered the same type of attempt?). We therefore resort to an indirect measure which nonetheless provides intuitive notions of attempt type. Specifically, we use the classification methodology of (Allen et al., 2020) that groups attempts using nonparametric clustering. This allows the “type of attempt” to be defined by the data, grouping placements together in ways that can allow for custom definitions of “similar” across levels without requiring any explicit definitions. To compare attempt types across groups, we applied a leave-one-out classification analysis where, for each participant, we formed probability distributions over the tool identities and spatial positions by all other members of their group and those from members of the other group, then calculated the relative likelihood that the tool placement for a given level was a member of the correct group. To investigate whether attempt types change from attempt to attempt, we formed clusters over tool spatial positions using all attempts from all participants within a level. Two consecutive attempts were considered a “switch” if each attempt came from a separate cluster.

To determine clusters for each different level, we aggregated all attempts across all participants, taking the [*x*, *y*] spatial positions of each attempt as variables but ignoring the specific choice of tool.^1^ We applied clusters separately for each participant group (children vs. adults, limb differences vs. no limb differences) but aggregated data across all participants from each group. Applying a Dirichlet Process mixture modeling package then gave us clusters for each level and group, as well as the probabilities that each attempt belonged to each cluster. We defined a “switch” as occurring if, for consecutive attempts, the cluster assigned as the highest likelihood was different for both of those attempts. Examples of discovered clusters for the data presented in Fig. 8 are shown in Fig. S6.

## Results

We will discuss in turn (1) the equating of basic motor abilities across groups, (2) participants’ overall performance metrics, (3) differences in cognitive styles that arise during solution finding, and (4) the detailed kinds of attempts each group made. For each section we will focus on the effect of embodiment but also note the effects of age where present.

### Basic motor abilities across groups

**Fig. 4:**
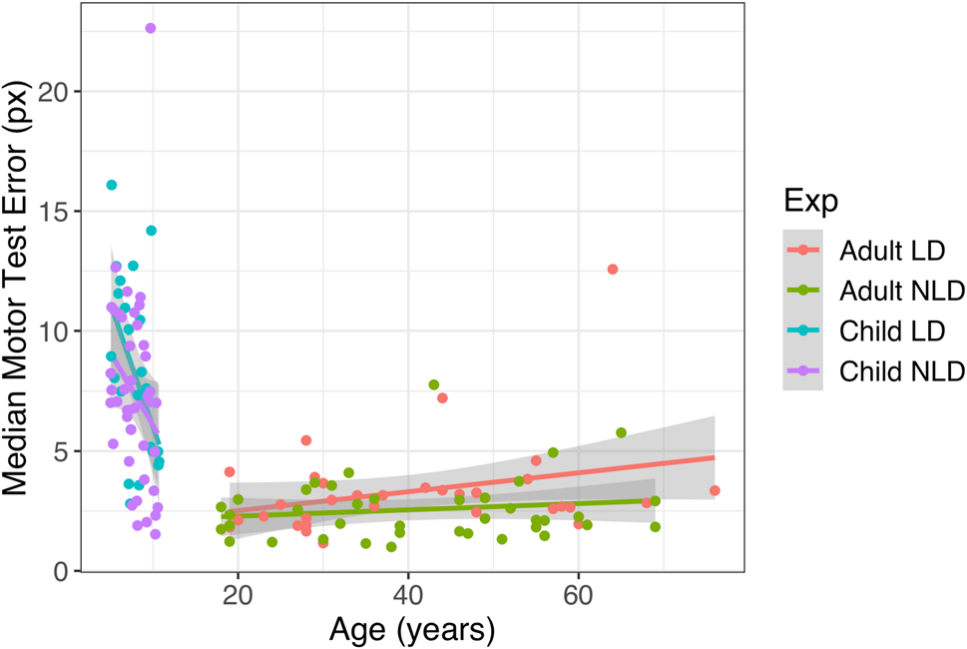
Reaction time on the motor task by participant age and group. Participants with limb differences were slightly *faster* on this task, suggesting that any differences in time to act or solve problems in the Virtual Tools game are not driven by differences in cursor control capabilities

We used the motor pre-test to examine whether there were group differences in cursor control which could affect performance on the Virtual Tools game. Children could control the cursor, with an average pixel error of 7.65*px* (95% CI=[6.74, 8.55]) and reaction time of 3.04*s* (95% CI=[2.67, 3.41]), albeit less accurately and more slowly than adults (error: 2.92*px*, 95% CI=[2.52, 3.31]; RT: 1.91*s*, 95% CI=[1.73, 2.09]). Exploratory analysis showed that children’s control improved linearly with age ($t(65) = 3.09,~p=0.003$, Fig. 4).

Importantly, individuals with and without limb differences performed comparably on both motor error and reaction time. While there was a difference in median click-time between participants with and without limb differences, participants with limb differences were slightly *faster* (by 356*ms*, 95% CI=[14, 698]; *F*(1,135) $=$ 4.15, $p =$ 0.044), though they clicked marginally further away from the target (by 0.79*px*, 95% CI=[$-0.14$, 1.72]; *F*(1,135) $=$ 2.78, $p =$ 0.098). There was no interaction found between age group and limb difference for either motor speed (*F*(1,134) $=$ 1.76, $p =$ 0.19) or error (*F*(1,134) $=$ 0.001, $p =$ 0.98). The differences found in motor control were relatively inconsequential for the Virtual Tools game – 0.79*px* additional error would have little effect on 600*x*600*px* game screens, and an extra 356 *ms* would be hard to detect with an average time between attempts of over 10 *s*. We therefore showed that both groups should have a level playing field for interacting with the Virtual Tools game.

**Fig. 5:**
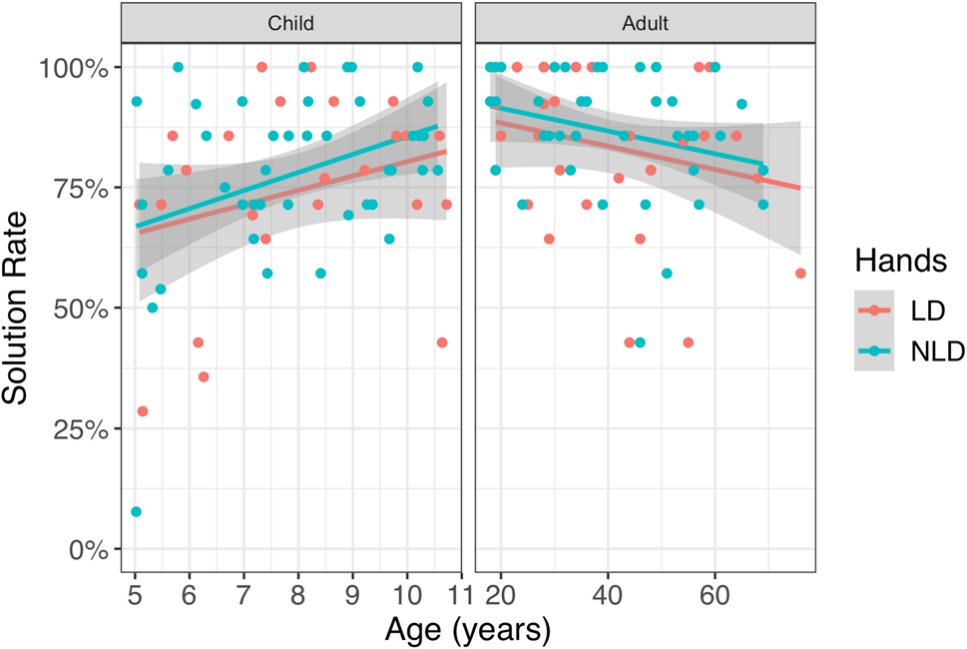
Solution rate (percentage of levels solved by each participant) as a function of age for children (*left*) and adults (*right*) with limb differences (LD) and with no limb differences (NLD). Grey areas represent standard error regions on the regression lines

### Performance metrics across groups

In the Virtual Tools game, we initially tested whether limb difference and age affected overall solution rates or time (in seconds) to reach a solution. We found gross differences in solution rates between children and adults (adults: 85%, children: 77%; $${x^2}(1)=$$ 11.20, $$p = $$ 0.0008; Fig. 5) but no effect of limb difference ($${x^2}(1) = $$1.21, $$p = $$ 0.27), nor any interaction between age and limb group ($${x^2}(1) = $$ 0, $$p =$$ 0.99). Exploratory analysis showed that age in years additionally predicted success ($${x^2}(2) = $$12, $$p =$$ 0.0025): while children’s solution rates improved with age (log-odds increase per year: 0.290, 95% CI =[0.038, 0.543]), adults’ worsened (log-odds decrease per year: 0.290, 95% CI =[0.006, 0.053]).

**Fig. 6:**
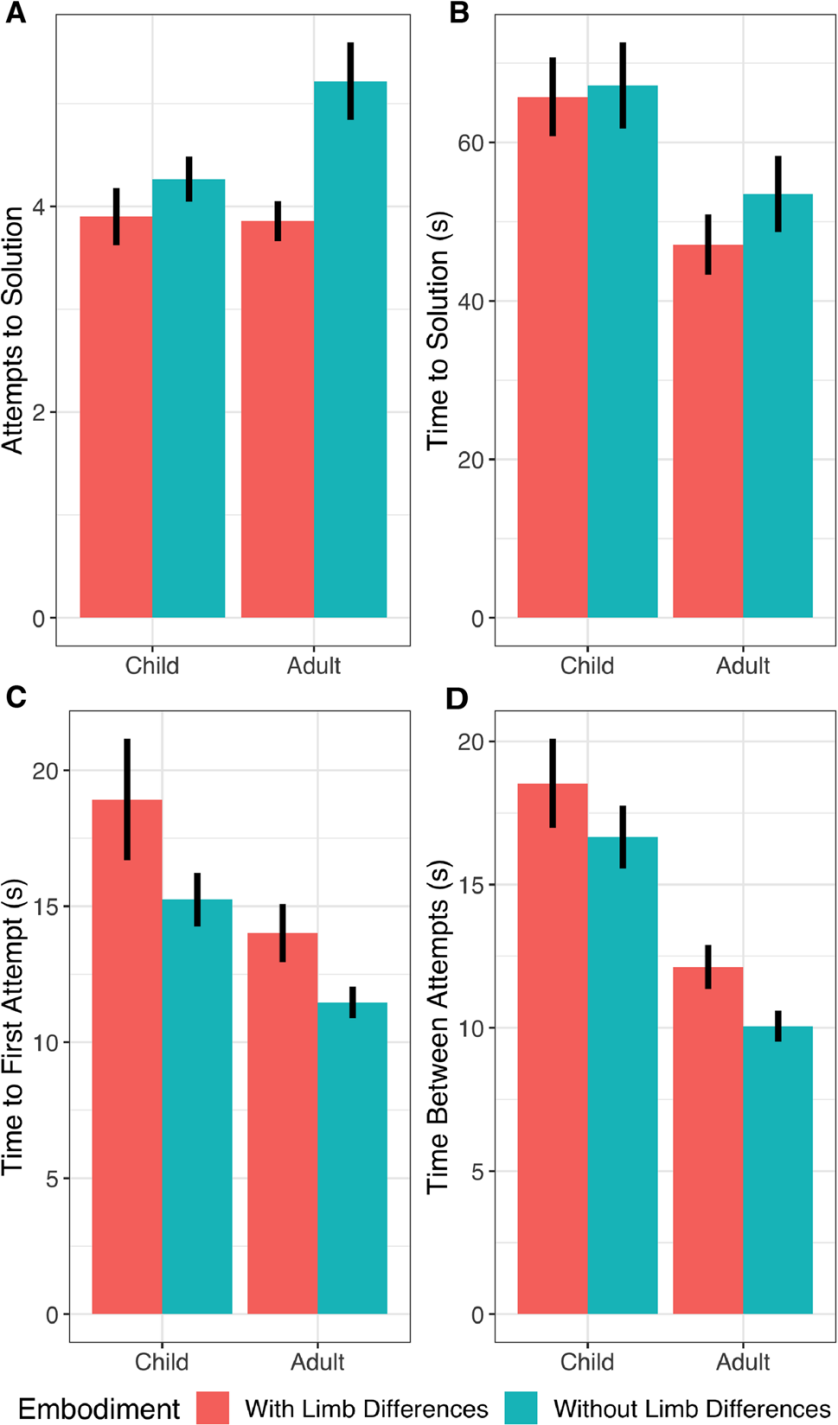
The efficiency of finding solutions measured by number of attempts (**A**), time to solution as measured in seconds (**B**), time to first attempt (**C**), and time between attempts (**D**). Means with standard errors are shown. Participants with and without limb differences did not reliably differ on time to solution but participants with limb differences solved the levels in fewer attempts, and took more time until the first attempt and between attempts

On time to solution, children were similarly slower than adults (${x^2}(1) ={40.0}, p = {2.6*10^{-10}}$; Fig. 6B) but we found no effect of limb differences (${x^2}(1) ={0.41}, p = {0.52}$), nor was there an interaction between limb group and age group (${x^2}(1) = {0.22}, p = {0.64}$). Adults slowed down with age (average additional 1.16*s* per year, 95% CI=[0.87, 1.46], (${x^2}(1) ={59.4}, p = {1.3*10^{-14}})$, while children non-significantly sped up (2.64*s* less per year, 95% CI=[$-1.63$, 6.90], ${x^2}(1) ={1.47}, p = {0.23}$).

Thus we found that while age causes noticeable changes in overall performance on the Virtual Tools game, limb differences do not. Nonetheless, participants might achieve similar overall levels of performance in different ways. We therefore next considered whether participants with or without limb differences might demonstrate distinctions on the more detailed performance metrics specified in Allen et al. (2020).

### Cognitive styles in solution finding

We investigated whether there was a difference in the number of attempts that participants with and without limb differences took to solve each level. Participants with limb differences (LD) took fewer attempts on average to come to a solution than the participants with no limb differences (NLD; $$83.9\%$$ of the attempts, 95% CI=[$$72.2\%$$, $$97.6\%$$]; $${x^2}(1) = $$ 5.19, $$p =$$ 0.023; Fig. 6A). Conversely, they took more time for each attempt, including taking more time before the first attempt (3.79*s* more, 95% CI = [1.99, 5.59]; $${x^2}(1) ={17.0}, p = {3.7*10^{-5}}$$; Fig. 6C), and between all subsequent attempts (2.80*s* more on average, 95% CI = [1.51, 4.10]; $${x^2}(1) ={17.9}, p = {2.3*10^{-5}}$$; Fig. 6D). Again, we found differences by age, with children taking fewer attempts (89$$\%$$ of the attempts as adults, $${x^2}(1) = {6.51}, p = {0.011}$$); more time to the first attempt (6.1*s* more, $${x^2}(1) ={13.4}, p = {0.00025}$$); and more time between attempts ($${x^2}(1) ={41.7}, p = {1.1*10^{-10}}$$) but no evidence for an interaction between age and limb differences for any of these measures (number of attempts: $${x^2}(1) = $$ 2.07, $$p =$$ 0.15; time to first attempt: $${x^2}(1) = {0.10}, p = {0.76}$$; time between attempts: $${x^2}(1) ={0.16}, p = {0.69}$$).^2^

Together, these results suggest that individuals born with limb differences learn a different cognitive style for physical problem-solving: they learn to spend more time considering the problem and less on gathering information from their attempts. While it is not possible to conclusively determine why individuals with limb differences spent more time on each attempt (i.e., it could relate to initiation costs (Khalighinejad et al., 2021) or habit formation from prior experience (Wong et al., 2017)), this extra consideration time was connected at the group level to fewer overall actions being needed to solve the problem. We therefore tentatively interpret the difference in reaction time as “thinking” – some internal computation that supports solving problems with fewer numbers of attempts.

### Attempt types

We first investigated whether there were differences in the types of first attempts taken by participants with and without limb differences, using the methodology described in the Methods: Analysis section to classify whether the types of attempts made by participants with limb differences better matched those of other participants with limb differences than those without, and vice versa. If this measure is on average reliably above chance on a level, this suggests that the two groups are beginning their solution search in different ways. However, we did not find statistically reliable effects. For qualitative differences between groups see Fig. 8, and Section S10 of the Supplemental Material for further details. For direct analyses of “error” types (what kinds of errors each group makes), please refer to Section S6 of the Supplemental Material.

**Fig. 7:**
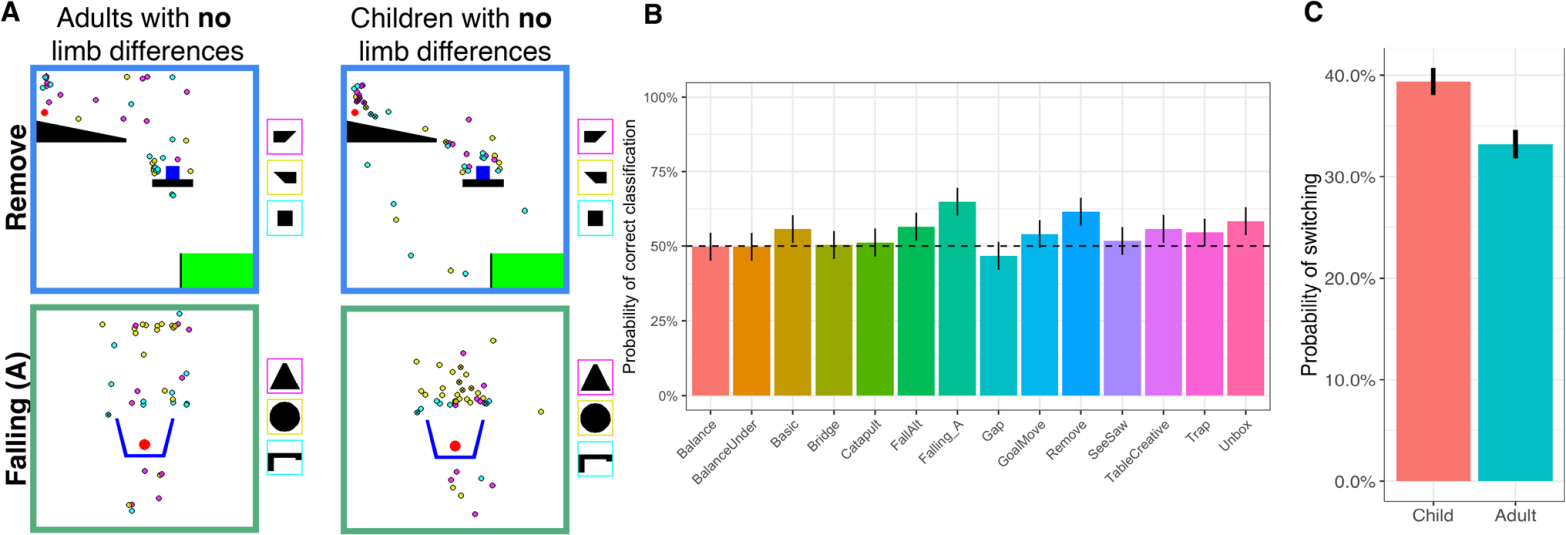
Comparing tool placements across children and adults born with two hands. Bar plots show means and standard errors. (**A**) Examples of first attempts for both adults and children without limb differences on two levels. Each point shows an individual participant’s attempt, with the position being where they placed the tool, and the color representing which tool they chose (tools shown in colored boxes to the right of each level). (**B**) We tested whether we could classify participants’ age group based on attempt type for each level. The dashed line represents 50$\%$ (chance). (**C**) The likelihood that children would “switch” attempt types. Please see Methods: Analysis and Section S5 in the Supplemental Material for details on how attempt type switching was measured

Using the same methodology, we found that across all levels, children’s and adults’ first attempt types can be differentiated (Fig. 7B; note that in this cluster analysis comparing age groups, we cannot jointly model the effect of limb differences and age, so we report analyses separately for each group; NLD: $t(84) = {4.91} p = {4.5^*10^{-6}}$; LD: $t(57) =$ 3.05, $p =$ 0.0035). This analysis suggests that children are starting with some sort of different action than adults but it cannot tell us exactly what differs.

To explicitly test for what differs, we measured whether we could find differences between the two groups using more structured analyses of error types (e.g., using an unworkable tool, placing the tool above vs. below the correct object) but could not find reliable differences (see Section S6). However, we did find that adults on average placed their tool vertically farther from the object than children (average vertical distance in children: 74*px*, adults: 91*px*; $${x^2}(1) = $$ 5.20, $$p = $$ 0.023), which is likely driving some of the differences. Nonetheless, we cannot make strong claims about why children and adults differ along this vertical dimension – e.g., children might just be more conservative, might be worse at understanding how gravity transfers into this environment, or might differ for a variety of other reasons. Furthermore, given the small absolute difference in vertical positions between groups, more work may be needed to understand this result.

**Fig. 8:**
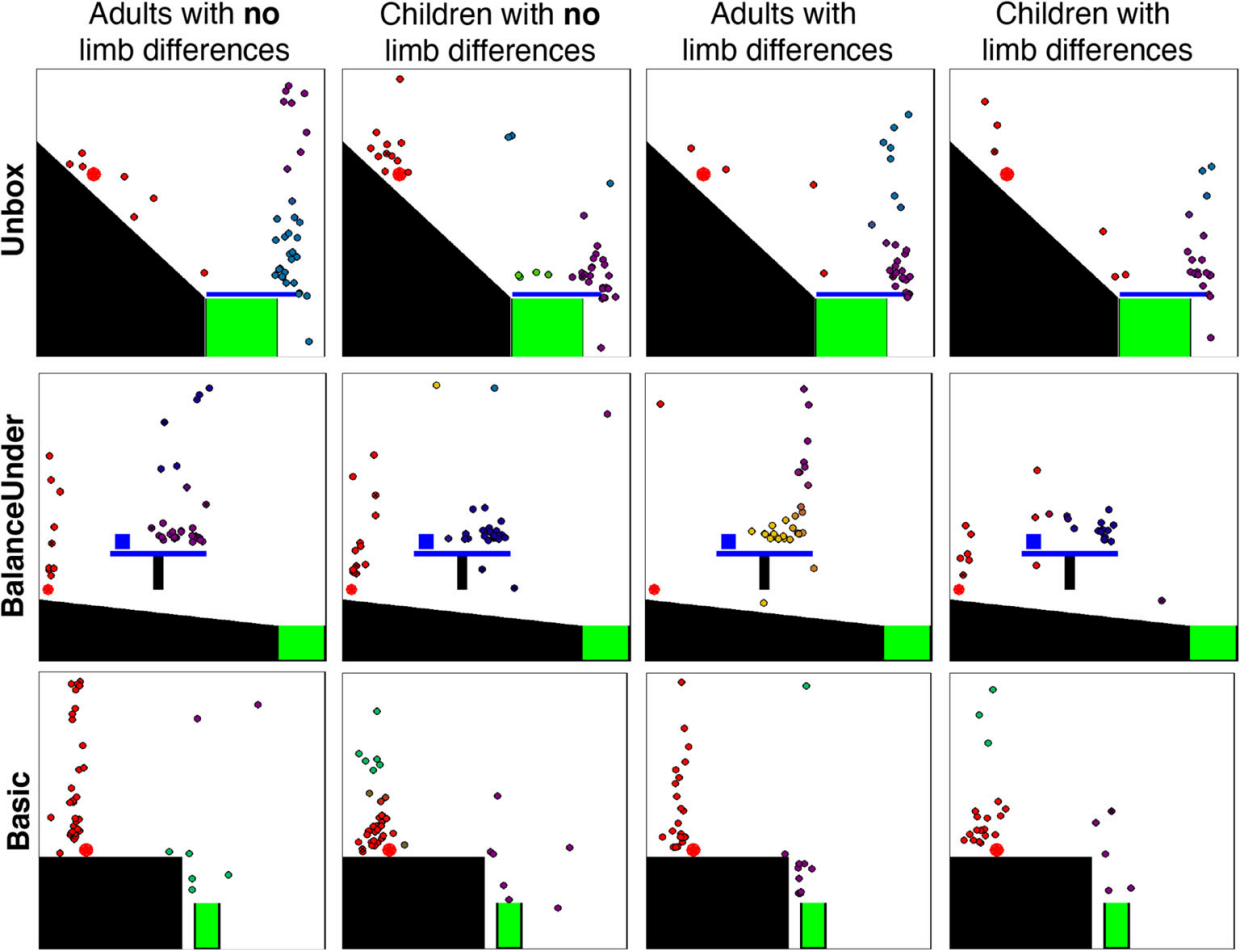
Comparison of tool placements on the first attempt for children and adults with and without limb differences. Each point represents an individual participant’s first attempt, with the position being where they placed the tool, and the color representing membership in “attempt type” clusters determined by Dirichlet Process Mixture Modeling over tool positions. Points of the same color therefore represent participants who chose first attempts belonging to the same attempt type

Since children are often more exploratory than adults (Gopnik et al., 2001), we also tested whether they might be more likely than adults to try new attempt types over the course of a single level. Children’s and adults without limb differences’ attempts were used to form clusters describing different tool “attempt types” (see Methods: Analysis for details), and we assigned each attempt to one of these (Fig. 8). Within a level, children were more likely to try new attempt types than adults (children: $$39\%$$ attempt type switches, adults: $$33\%$$; $${x^2}(1) = $$10.2, $$p = $$ 0.0014), suggesting that their lower solution rates might be due to either increased exploration of inefficient attempt types, or giving up on promising attempt types early. Using a more structured measure of switching based on the same error types above (a “switch” being defined as changing the qualitative spatial position of the tool relative to an object in the scene, or switching which object in the scene to interact with), we similarly found a difference in switching behavior, with children switching $$56\%$$ of the time, and adults $$47\%$$ of the time ($$p=0.0009$$). While statistically significant, given the relatively small absolute difference in switching behavior, more work may be needed to investigate this result further.

## Discussion

We used a virtual physics problem-solving game to study how growing up in a different body affects a high-level cognitive task unrelated to body or hand representations. We found minimal differences in the specific kinds of actions used by individuals with and without limb differences, suggesting that fundamental aspects of physical problem-solving are not dependent on similar kinds of manipulation experience. However, we also found that individuals with limb differences, regardless of age, spent more time considering virtual physical problems, and took fewer attempts to find solutions. While congenital limb difference is not directly associated with cognitive differences (see Section S2.1 in the Supplemental Material), growing up with a limb difference may cause cascading effects on many aspects of development, relating to different opportunities to interact with the environment. For example, children with limb differences may be unable to solve problems by imitating their parents or peers, or may face challenges in using tools designed for two hands. In either case, they might come to appreciate the value of thinking more about solutions to physical problems before acting, and over time this could grow into a general cognitive style for interacting with the world. What is striking is that this learned cognitive style extends to a task in which action possibilities are equated across groups – indeed, individuals without limb differences were not slower to control the cursor in our task (see also (Maimon-Mor et al., 2021)).

We call these differences in “cognitive styles,” as they fall under the definition of “consistent individual differences in preferred ways of organizing and processing information and experience” (Messick, 1976). However, while much of the previous research on cognitive styles has focused on how these individual differences might arise due to personality differences (Kozhevnikov, 2007), here we suggest that an alternative driver of different cognitive styles might be the body that people inhabit, which in turn affects the costs of interacting with the world.

Cognitive styles are often thought to be learned through experience, similarly to studies of motor learning in adults (Huang et al., 2012). The difference here is that the target of learning is not the motor plan itself but *when to deploy* those motor plans. While people’s information sampling has been shown to be sensitive to the costs of obtaining that information (Juni et al., 2016; Jones et al., 2019) and motor cost manipulations have been shown to affect the efficiency of motor reaching actions (Summerside et al., 2018), it has not previously been shown that motor differences directly affect the cognitive styles that people employ.

Our findings also bridge two different approaches to understanding human tool use. Tool use is theorized to be supported by specific sensorimotor knowledge of tool manipulation under the “manipulation-based” (embodied cognition) approach (Buxbaum and Kalénine, 2010; Gonzalez et al., 1991; van Elk et al., 2014), or generic physical knowledge under the “reasoning-based” approach (Allen et al., 2020; Osiurak and Badets, 2016). These theories have produced suggestions that there are distinct cognitive systems supporting different kinds of tool knowledge (Orban and Caruana, 2014; Goldenberg and Spatt, 2009). However, our results suggest a connection between the two systems: by its virtual nature and novel objects, the Virtual Tools game must rely on reasoning-based systems for tool use, yet we find that manipulation capabilities affect this reasoning. Thus we suggest that the development of the reasoning-based system is grounded in the embodied way that we interact with the world.

Our results extend existing knowledge about children’s problem-solving and tool use. Children can use and select known tools by 2–3 years old (Keen, 2011) but do not reliably innovate new tools until 8–9 years (Beck et al., 2011), perhaps due to its increased cognitive demands (Rawlings & Legare, 2020). Children’s performance in our game is likely driven by these same planning and attentional skills, which underpin a useful balance between exploration and exploitation in a large solution space (Gopnik, 2020). Specifically, while children at this age can avoid perseveration (Rawlings & Legare, 2020; Cutting et al., 2011), our results suggest over-exploration may be an issue, as children switched attempt types more often than adults. The central difficulty with tool innovation and other physical problem-solving tasks at this age may be the need to search through large solution spaces, where children’s propensity for exploration (Oudeyer and Smith, 2016; Gopnik, 2020) comes at the cost of short-term gains in solution-finding.

Our findings suggest new interactions between embodiment, development, and cognitive styles and raise important questions for future work. The present study focused on a single task - the Virtual Tools game - because it is similar to manipulation tasks while still not requiring manipulation directly. Thus, we expected cognitive styles learned from lifelong differences in action costs and possibilities to carry over into this task, while still providing an equal playing field for all our participants.

One might ask how broadly these differences in cognitive styles would be expected to generalize, e.g. across domains and environments. Some previous work indirectly supports the notion of a broad effect across motor-related tasks. For example, individuals with a limb difference responded more slowly in motor planning (Philip et al., 2015) and hand laterality judgements (Maimon-Mor et al., 2020). These findings were previously interpreted as a consequence of having fewer available resources to accumulate evidence for tasks related to judgements about hands. However, in light of the present findings, and because the previous observed effects were also found for the intact hand, these results can be interpreted as a *generally* greater reliance on planning before acting for this population. Yet, these tasks all contain a visuospatial and motor component, so it is still unclear how broad this effect is, e.g., whether it transfers to abstract logical reasoning tasks or social interactions. It is also difficult to determine, based on our single study, whether the group differences we observed are directly or indirectly caused by different embodied experience considering the many developmental differences individuals growing up with a different body will experience. For example, life experiences for individuals born with different bodies may cause them to be generally more cautious, contemplative, or creative.

Thus, the current study provides a starting point for further investigation of how we learn to deploy our cognitive resources based on our embodied experiences. Being born with a different body does not change the fundamental ways in which people try to act on the world but it can change the styles they acquire in order to plan and act efficiently in their environments.

## Open Practices Statement

This study was not preregistered. The data and analyses have been made available on a permanent third-party archive https://github.com/k-r-allen/embodied_experience. The stimuli for the study can be found at https://sites.google.com/view/virtualtoolsgame and in the Supplemental Materials.

### Supplementary Information

Below is the link to the electronic supplementary material.

Supplementary file 1 (pdf 1187 KB)

## Data Availability

The data have been made available on a permanent third-party archive https://github.com/k-r-allen/embodied_experience. The stimuli for the study can be found at https://sites.google.com/view/virtualtoolsgame and in the Supplemental Materials.

## Footnotes

1 Tool orientation could not be controlled, so was not included as a variable

2 When testing for the effects of limb differences in children and adults separately, we found reliable differences in attempt timing in both children (first attempt: 4.13*s*, 95% CI = [0.96, 7.30], $${x^2}(1) = $$ 6.52, $$p =$$ 0.011; between attempts: 2.57*s*, 95% CI = [0.31, 4.84], $${x^2}(1) = $$ 4.96, $$p =$$ 0.026) and adults (first attempt: 3.29*s*, 95% CI = [1.32, 5.26], $${x^2}(1) = $$ 10.7, $$p =$$ 0.0011; between attempts: 2.63*s*, 95% CI = [1.26, 4.01], $${x^2}(1) =$$14.2, $$p = $$ 0.00017). However, while we found that adults with limb differences use fewer attempts than those without ($$75.1\%$$ of the attempts, 95% CI = [$$59.1\%$$, $$95.4\%$$], $${x^2}(1) = $$ 5.49, $$p =$$ 0.019), children with limb differences took numerically fewer attempts than those without but this does not reach statistical significance ($$94.9\%$$ of the attempts, 95% CI = [$$78.2\%$$, $$115.0\%$$], $${x^2}(1) = $$ 0.29, $$p =$$ 0.59). Thus, while we can claim that participants with no limb differences overall took more attempts, we did not have enough evidence to discriminate whether this is because these differences are consistent across age groups, or whether the distinction grows through development.

## Code Availability

The code has been made available on a permanent third-party archive https://github.com/k-r-allen/embodied_experience.
