# Supplementary material for: Lifelong learning of cognitive styles for physical problem-solving: The effect of embodied experience
Source: Psychon Bull Rev. 2023 Dec 4;31(3):1364–75. doi: 10.3758/s13423-023-02400-4 (PMC11192818; doi:10.3758/s13423-023-02400-4)
Supplement: Supplementary file 1 — (pdf 1187 KB) [file 13423_2023_2400_MOESM1_ESM.pdf]

Supplemental Material

S1 Additional experimental details

S1.1 Virtual Tools Game

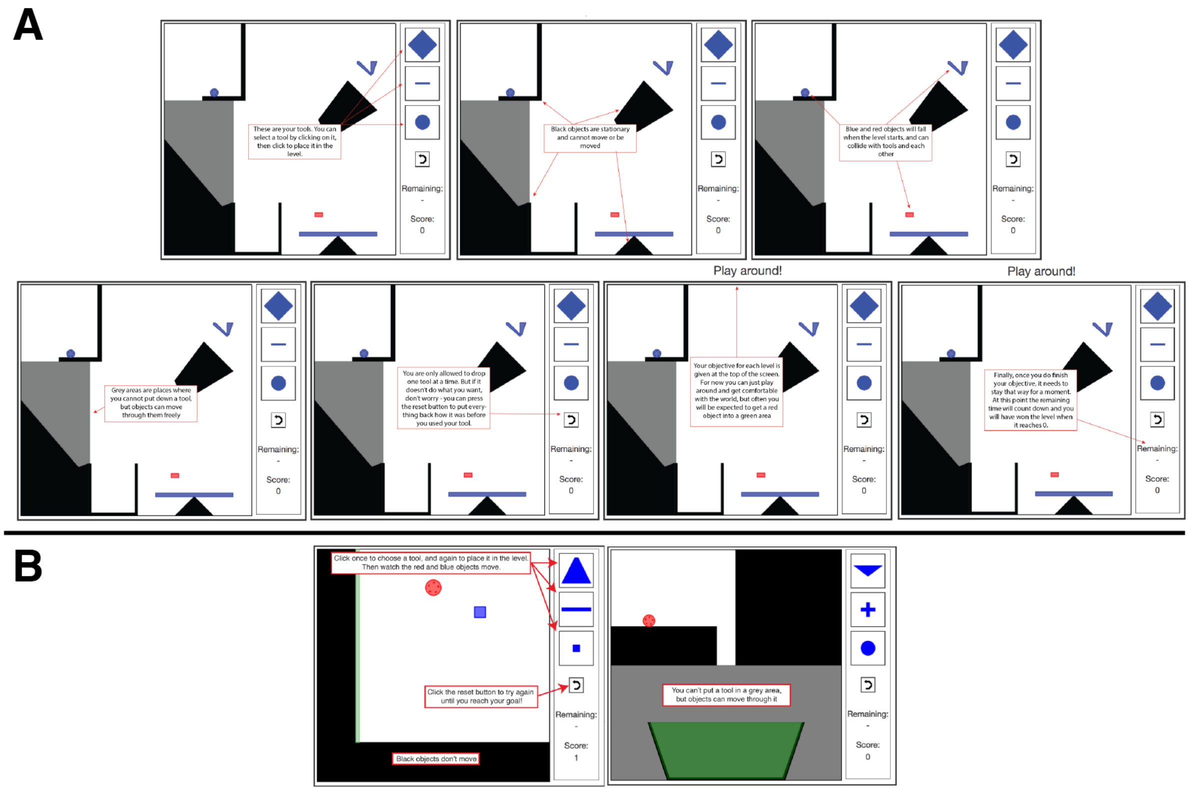

Figure S1. Instruction screens provided to **A.** adults and **B.** children.

The instructions provided to children and adults for the Virtual Tools game are provided in Figure S1. Introductory levels which must be solved (by both children and adults) but were not analyzed were the levels shown in the childrens' instructions.

S1.1.1 Solution maps for each level. To gesture towards the complexity of the different levels in the kinds of solutions they allow, we include here the "solution maps" for each of the 14 levels played by participants. These are color coded to indicate which pixels each of the different tools will solve the level in. The tools are color-coded as pink for the first object, yellow for the second, and cyan for the third.

S1.2 Motor pre-test

The motor pre-test ensured that participants with and without limb differences had equal abilities to interact with the computer system, and therefore should be equally able to control the Virtual Tools game. The motor-test is shown in Figure S3. Participants were required to click a central star before clicking a

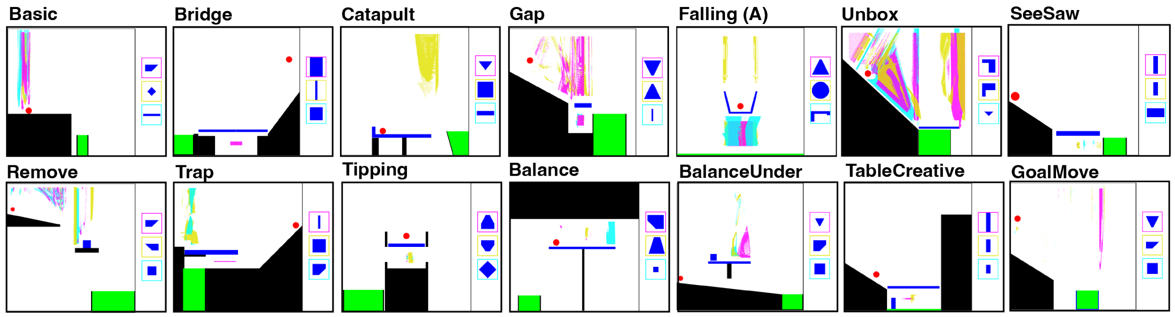

Figure S2. Solution maps for each of the 14 levels played by participants in the Virtual Tools Game. Each pixel is colored pink if the first available tool, placed there, solves the level, yellow if the second available tool does, or cyan if the third tool. As can be seen across levels, in most cases there are many possible solutions.

colored circle in the periphery (either 150 or 250 px from the center of a 600x600px screen). They completed 10 rounds of this procedure.

We presented median motor RT (our motor covariate) as Figure 4 in the main text, but see Figure S5 for median motor error (in pixels).

S2 Participant demographics

S2.1 Education levels

Adults were matched on education level, and we tested for similarities in cognitive capabilities by performing IQ tests on a subset of the children from both the LD and NLD groups. These tests were provided in a separate session from the main experiment. We assessed both Raven's matrices measures of spatial IQ, and BPVS as a measure of verbal IQ. 20/25 Child-LD and 34/43 Child-NLD participants were tested. All scores were within normal range (lowest for Ravens 85, highest 135, lowest for BPVS 75, highest 134. Ravens means: Child-LD: 113, Child-NLD: 111 for Ravens. BPVS means Child-LD: 105, Child-NLD: 108), and 2-sided unpaired t-tests show no evidence for differences between these populations (Ravens: $t(40) = 0.5,\ p = 0.6$, BPVS: $t(50) = 0.8,\ p = 0.4$).

S2.2 Demographic and interface information

Please see Table S1 for information on participant handedness as well as how they interfaced with the computer.

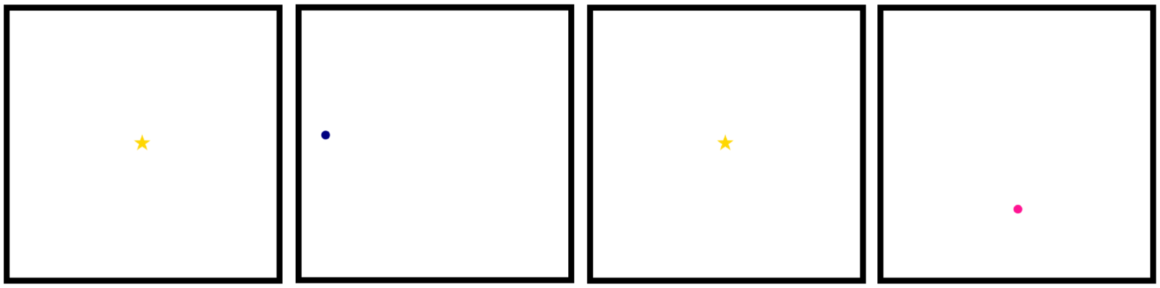

Figure S3. An example of two rounds of the motor pre-test. Participants clicked first the star, then a circle in the periphery, as quickly as possible.

Group	Mean Age (SD)	Handedness			Input Device		
		Left	Right	Ambi.	Mouse	Touchpad	Other
Adult-LD	40.7 (15.5)	0.45	0.55	0	0.61	0.36	0.03
Adult-NLD	41.2 (15.2)	0.08	0.92	0	0.55	0.45	0
Child-LD	7.91 (1.84)	0.36	0.64	0	0.32	0.68	0
Child-NLD	7.89 (1.73)	0.07	0.89	0.04	0.50	0.50	0

Table S1

Demographics summaries for participants. LD: limb differences; NLD: no limb differences

S2.3 Specific limb differences

For details on specific limb differences in the children we tested, please refer to Table S3. Adult participants had less variability in their limb differences. Of the 33 adult participants, one had a bilateral limb difference (with a missing right arm at the shoulder, and left arm at the elbow), and 32 had unilateral limb differences, including:

- 3 transhumeral
- 3 at the elbow
- 18 transradial (one participant has a small residual digit)
- 8 at wrist (one participant has 5 small intact digits; one has a short thumb and small, non-jointed digit; one has two residual digits)

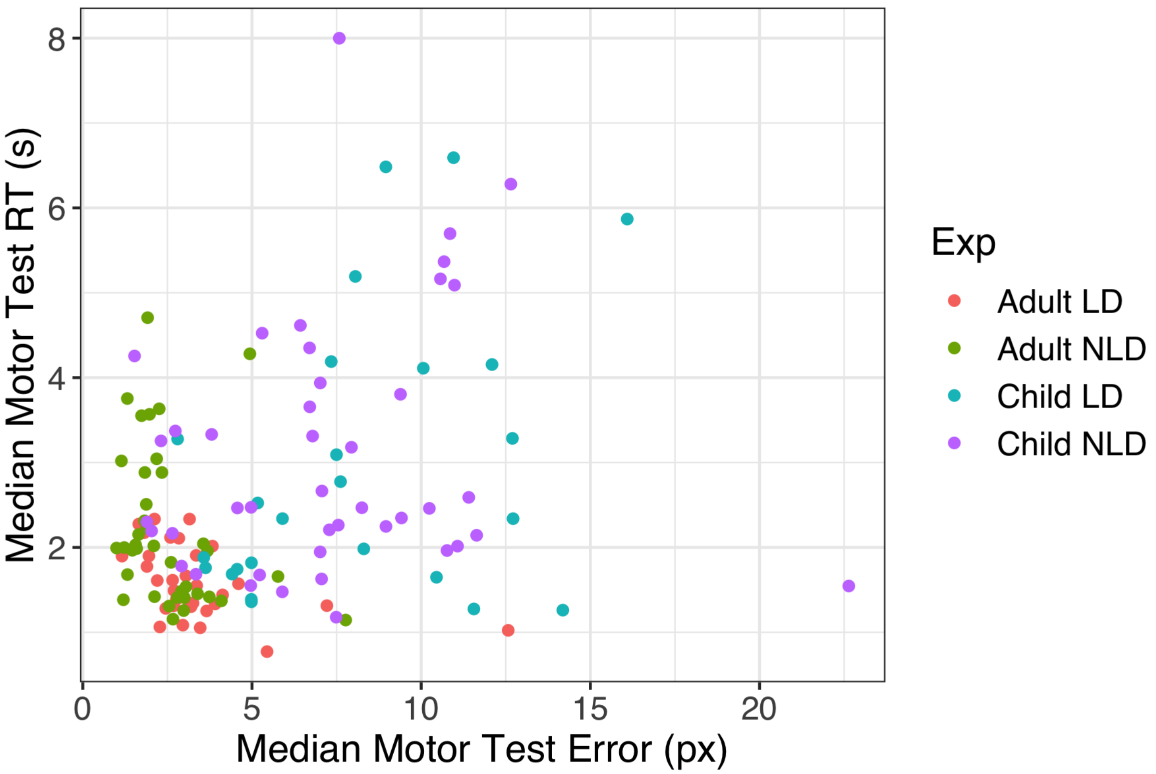

Figure S4. Comparison of Median Motor test reaction time with median motor test error for each participant across groups. LD = participant with limb differences, NLD = participant with no limb differences. Correlation is reasonably high, $r = 0.34$, so we use only the median motor test error as a covariate throughout analyses.

S3 Exclusions

We excluded two adults with limb differences from analysis as they were amputees rather than having congenital limb differences. We also excluded results from two two-handed children: one due to a data recording error, and one who provided unreliable motor test data due to continuously clicking rather than attempting the task.

Adult participants were given 15 different levels to solve: the 14 shown in Fig 2C, and one additional level: Spiky (see Allen et al., 2020). Because of the low solution rate of adults on this level (18%), we were concerned that it might frustrate children and cause attrition, so only had children play the other 14 levels. To keep the groups matched, we therefore removed the Spiky level from analysis.

S4 Analysis details

We modeled all statistical analyses as (generalized) linear mixed effect models using the 'lme4' package in R (Bates, Mächler, Bolker, & Walker, 2015). We treated accuracy as a binomial response, time

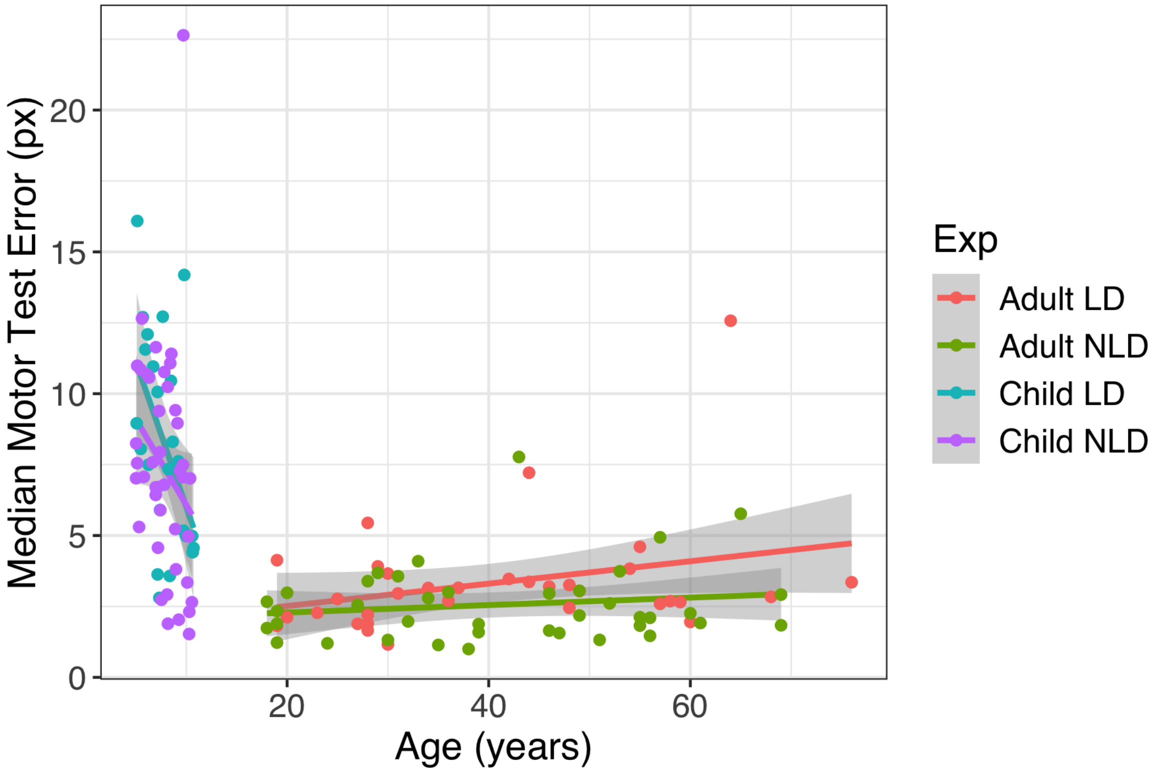

Figure S5. Median motor test error in pixels as a function of age. Children improve significantly as they age, while adults' performance is unaffected. No interaction was found between age and embodiment group (($F(1, 134) = 0.001,\ p = 0.98$)).

measures as having Gaussian error, and attempts as a Poisson process (using the number of non-solution attempts as the dependent variable so that we could observe zero-attempt outcomes). In all models, we assumed random intercepts for participants and levels.

Additionally, we prespecified two covariates for all of our analyses. We used the median motor test response time as a covariate, as we had hypothesized that motor facility might cause better performance. We selected response time instead of error because the two measures were correlated ($r = 0.34$), and in pilot analyses we found that adding a second motor measure explained very small amounts of additional variance in performance over just a single measure. Finally, because we assumed that there would be large effects of age on performance, for all analyses testing the difference between participants with and without limb differences, we included age as a covariate, allowing its effects on performance to differ for children and adults (treated as an age by child/adult interaction).

S5 Attempt clustering procedure

To measure exploratory behavior, in the Results we looked at how likely participants were to "switch" strategies between attempts. Since strategies are hard to quantify, we used a nonparametric clustering package implemented in Python's SciPy package (Virtanen et al., 2020) to discover strategy clusters from participant data. For each different level, we aggregated all attempts across all participants, taking the $[x, y]$ spatial positions of each attempt as variables, but ignoring the specific choice of tool. We applied clusters separately for each participant group (children vs. adults, limb differences vs. no limb differences), but aggregated data across all participants from each group. Applying the Dirichlet Process mixture modeling package then gave us clusters for each level and group, as well as the probabilities that each attempt belonged to each cluster. We defined a "switch" as occurring if, for consecutive attempts, the cluster assigned as the highest likelihood were different for both of those attempts. Examples of discovered clusters for the data presented in Figure 8 are shown in Figure S6.

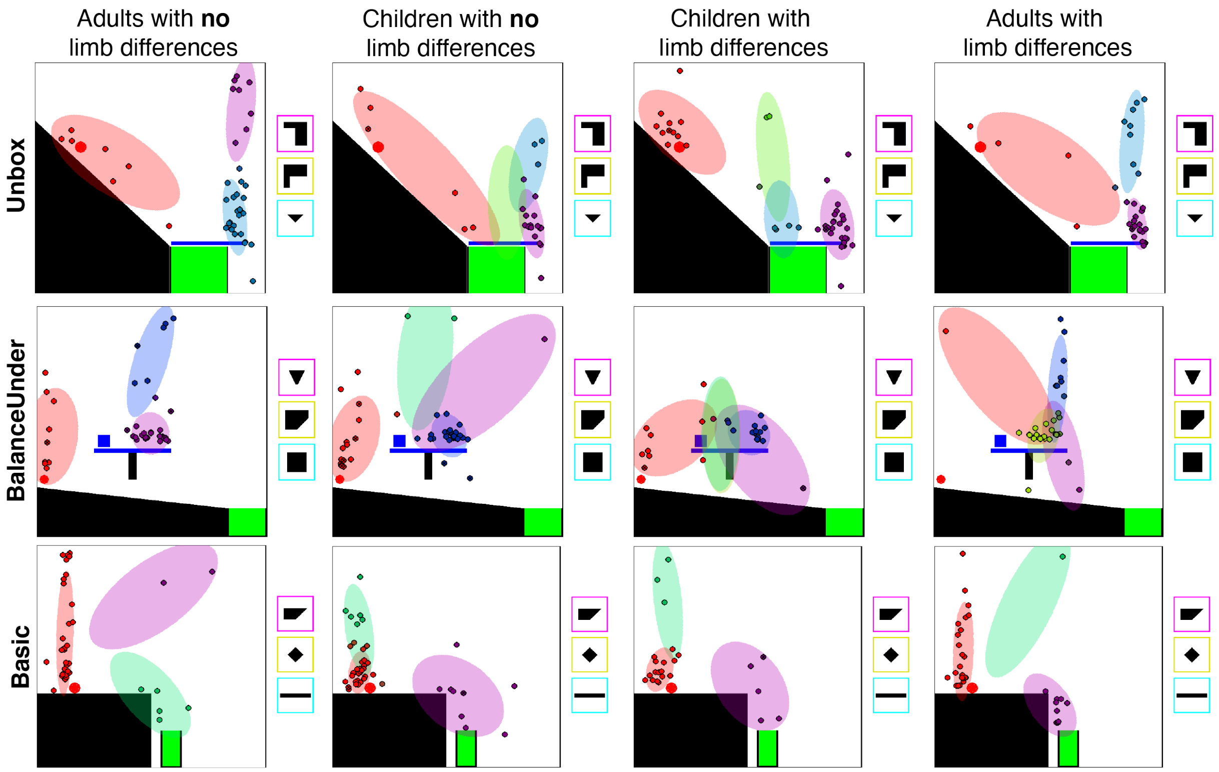

Figure S6. Examples of discovered strategy clusters (shown as semi-transparent ovals) with participants' first attempts overlaid as colored points. The color of each point represents the highest likelihood cluster for that attempt.

S6 Exploratory analyses of error types

In order to test for differences in the types of errors that our participants made, we attempted two exploratory analyses to test whether limb differences or age would have an effect on either (1) whether people used the wrong tool, and (2) the particular type of error people made.

Testing for whether people used the incorrect tool was difficult, as our Virtual Tools levels were designed to be solvable in multiple ways (albeit easier with some tools than others). In only 4 of 14 levels was there actually a tool that could never be used to accomplish the goal; thus our analysis was limited to those four levels (Bridge, Catapult, GoalMove, TableCreative). We analyzed this as the probability of selecting the incorrect tool on each action (using a binomial link function), and tested whether there was an effect of limb differences, age category, or age in years, on this probability (again using motor response as a covariate and participants and levels as random effects). We find that there is no evidence of impact of any of our primary independent variables on tool error rate ($\chi^2(4) = 5.3,\ p = 0.26$), and no individual factor or interaction reached statistical significance. However, because we could only analyze a limited subset of the data, we do not believe this provides strong evidence *against* there being differences.

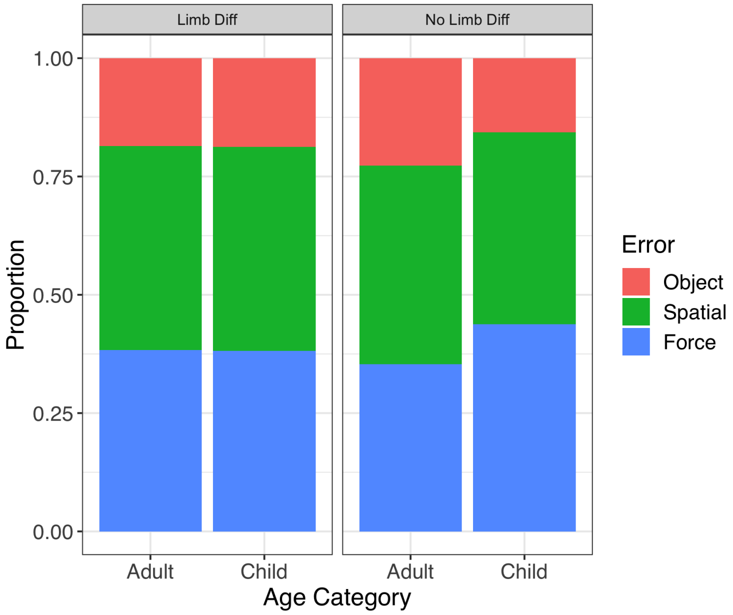

Figure S7. Proportion of types of errors made, split by age category and limb differences.

We also tested the type of errors our participants made, classifying them into one of three types. (1) Object errors: did people place the tool in a location that would not contact an object that would solve the goal? (2) Spatial errors: did people choose a placement such that the tool would contact an object that would solve the goal, but placed in the incorrect spatial relationship to where it needed to go (e.g.,

dropping a tool to hit a ball to the left rather than the right, or dropping a tool over an object instead of supporting it from below)? (3) Force errors: did people place the correct tool in the correct qualitative spatial location, but in such a way that it would not impart enough force to accomplish the goal (e.g., hitting a ball too lightly)? See Figure S7 for the distribution of these errors. Because there is an ordering to these error types, we modeled this as an ordinal logistic function, using the same explanatory variables and random effects as the incorrect tool analysis above. We find that the variance this model overall explains barely touches on statistical significance ($\chi^2(4) = 9.48,\ p = 0.050$), but it is not clear what is driving this effect: no individual term or interaction is statistically significant after correcting for multiple comparisons, though the term that gets closest is an interaction between the child/adult indicator and age in years ($z = 2.56$, $p_{corrected} = 0.055$), suggesting that younger children and older adults are less likely to make force errors, and more likely to make spatial or object-based errors. While this does align with our findings about overall solution rates (where younger children and older adults perform worse), due to the exploratory nature of this analysis and weak evidence from the statistics, we do not want to make any strong claims about this relationship; however, it does provide an interesting avenue for follow-up studies that could specifically test this effect.

S7 Analysis excluding covariates

Because we expected that age and motor capabilities would have strong effects on task performance, we used both continuous age measures and performance on the motor pre-test as covariates when testing for the effect of limb differences. However, because this experiment does not involve random assignment of participants to this dependent variable, we can and do find differences (albeit small) in these covariates across groups with and without limb differences. We therefore have additionally analyzed all primary measures *without* covariates to determine the effect of this analysis choice, and find qualitatively identical results, as reported in detail below. While there is a difference between children and adults on overall solution rate ($\chi^2(1) = 12.0,\ p = 0.00053$) and time to solve ($\chi^2(1) = 13.9,\ p = 0.00019$), there remains no effect of limb differences on either (solution rate: $\chi^2(1) = 1.3,\ p = 0.256$, solution time: $\chi^2(1) = 0.06,\ p = 0.803$), nor is there an interaction between limb differences and age group (solution rate: $\chi^2(1) = 0.00,\ p = 0.944$, time to solve: $\chi^2(1) = 0.46,\ p = 0.498$).

We still find that participants with limb differences take fewer attempts ($\chi^2(1) = 5.88,\ p = 0.015$) but more time thinking before taking their first attempt ($\chi^2(1) = 7.00,\ p = 0.0081$) and between subsequent attempts ($\chi^2(1) = 4.68,\ p = 0.031$, though no interaction between limb differences and age group (number of attempts: $\chi^2(1) = 2.21,\ p = 0.137$, time to first attempt: $\chi^2(1) = 0.286,\ p = 0.593$, time between attempts: $\chi^2(1) = 0.01,\ p = 0.915$). Similar to the analysis with covariates, adults take less time

to the first attempt ($\chi^2(1) = 13.09,\ p = 0.0003$) and between attempts ($\chi^2(1) = 42.9,\ p = 5.6 * 10^{-11}$).

S8 Controlling for possible moderators

In order to validate our choice of analysis, we explored whether any of a set of moderators might have driven any of the results we report in the paper. These moderators were not expected to have an impact on the results, but were tested in exploratory analysis to check that assumption. We specifically test (1) participant gender, (2) the type of device participants used to control the game (mouse or touchpad), (3) whether participants were dominantly left- or right-handed, and (4) for the DL participants, what kind of prostheses they used. While we find possible impacts of these variables on overall performance (as detailed in the next paragraph), in no cases do we find any interactions between these variables and embodiment, suggesting that they should not impact the main results of the paper.

Gender had a main effect on solution rate ($\chi^2(1) = 9.88,\ p = 0.0017$), with males slightly outperforming females (83% vs 77%), but no interaction with embodiment ($\chi^2(1) = 0.53,\ p = 0.47$). We also found a small effect of gender on the first attempt time (males: $13.8s$, females: $15.2s$, $\chi^2(1) = 5.56,\ p = 0.018$), but again no interaction with embodiment ($\chi^2(1) = 0.75,\ p = 0.39$). There were no other main effects of gender or interactions between gender and embodiment for any other performance metrics. Device type had a main effect on time to solution ($\chi^2(1) = 7.96,\ p = 0.0048$), with participants using a mouse solving the levels slightly faster than participants using a touchpad ($53.2s$ vs $63.8s$). However, we found no interaction between device and embodiment ($\chi^2(1) = 0.47,\ p = 0.49$), nor did we find any other main effects of input device type. We found no effect of hand laterality on any of our dependent variables. We similarly find no main or interaction effects for prosthesis use on any of our performance measures (all $ps > 0.12$).

S9 Analysis of all levels

In the main body of the text, we studied the impact of age and embodiment on how participants solved *successful* levels in order to avoid measuring effects of persistence or motivation. In order to test the effect of this analysis choice on our results, we additionally ran our main analysis including all levels. We find that in general, there is little difference between the success-conditioned and all-levels analyses, though one statistical test (overall number of attempts by embodiment) crosses from statistically significant to barely not statistically significant. Based on exploratory analysis, this appears to be driven by differences in perseveration between groups, with participants with limb differences persisting marginally more than those without. We discuss this difference and report all statistics below for transparency.

S9.1 Number of total attempts

While we found that participants with limb differences used fewer attempts to get to a solution than participants without, the effect of embodiment on number of total attempts is only marginally significant (participants with limb differences taking 88.1% of attempts of those without on average, 95% CI=[86.1%, 102.0%]; $\chi^2(1) = 2.92,\ p = 0.088$).

We ask whether any differences in this analysis might be a function of differences in motivation, and so measure persistence as the ratio of total attempts (regardless of success) to the number of attempts taken on successful levels. We find that by this definition, participants with limb differences were numerically more persistent (adults: 145%, children: 147%) than participants with no limb differences (adults: 132%, children: 136%), though this difference was only marginally significant ($F(1, 135) = 3.80,\ p = 0.053$). Because of the marginal significance, we do not make strong claims about the persistence of LD vs. NLD participants, but this numerical difference is likely what drives the reduction in the measured effect of embodiment on total attempts.

S9.2 Other results

Time to solution: similar to the success-conditioned analysis, we find that children take more time than adults ($\chi^2(1) = 37.8,\ p = 7.8 * 10^{-10}$), that there is no reliable effect of embodiment ($\chi^2(1) = 0.37,\ p = 0.55$), and that there is a difference by continuous age ($\chi^2(2) = 43.8,\ p = 3.1 * 10^{-10}$). *Time to first attempt:* just as with the success-conditioned results, we find that participants with limb differences took more time than participants with no limb differences until their first attempt ($4.12s$ more, 95%CI = [2.20, 6.03]; $\chi^2(1) = 17.7,\ p = 2.6 * 10^{-5}$), and that this differs by age ($\chi^2(1) = 12.4,\ p = 0.00043$), but no evidence of an interaction between age and embodiment ($\chi^2(1) = 0.32,\ p = 0.57$). *Time between attempts:* similar to the success-conditioned analyses, we find that participants with limb differences spend more time thinking than participants with no limb differences between attempts ($2.33s$ more, 95%CI = [1.04, 3.63]; $\chi^2(1) = 12.5,\ p = 0.00041$), and that this differs by age ($\chi^2(1) = 43.5,\ p = 4.2 * 10^{-11}$), but no evidence of an interaction between age and embodiment ($\chi^2(1) = 0.04,\ p = 0.84$).

S10 Analysis of differences between LD and NLD attempt types

Overall, we found a trend towards being able to classify participants with and without limb differences (mean adult classification accuracy: 51.4%, 95% CI=[49.9, 52.9], $t(71) = 1.91,\ p = 0.060$; mean child classification accuracy: 52.3%, 95% CI=[49.5%, 55.1%, $t(70) = 1.62,\ p = 0.109$), but found only one level where the confidence interval on the estimated classification probability exceeded chance for both

children and adults (see Table S4): BalanceUnder. This level requires preventing objects from falling by placing a tool as a counterweight to another object, which might be a strategy the participants with limb differences used more often in daily life, as they are have to rely on their residual arm (which is shorter than their intact arm) when manipulating objects bimanually. However, given that the estimated classification probability is not far from chance and that we cannot reliably classify attempts in other levels that rely on balancing, future work would need to investigate particular differences in strategies learned from interaction with the environment.

ID	Limb Difference				Dominant Limb	Used Limb	Prosthesis	
	Left	Pincer	Right	Pincer			Fitted	Use
1	Intact	Yes	Absent below elbow	No	L	Intact	Yes	1
2	Absent above elbow	No	Intact	Yes	R	Intact	No	-
3	Absent above elbow	No	Intact	Yes	R	Intact	Yes	2
4	Absent below elbow	No	Intact	Yes	R	Intact	Yes	2
5	Absent below elbow	No	Intact	Yes	R	Intact	Yes	2
6	Absent below elbow	No	Intact	Yes	R	Intact	Yes	1
7	Intact	Yes	Absent below elbow	No	L	Intact	Yes	1
8	Intact	Yes	Absent below elbow	No	L	Intact	No	-
9	Absent below elbow	No	Intact	Yes	R	Intact	Yes	2
10	Absent below wrist	No	Intact	Yes	R	Both		
11	Absent below elbow	No	Intact	Yes	R	Intact	Yes	2
12	Absent below elbow	No	Intact	Yes	R	Intact	Yes	1
13	Intact	Yes	Absent below elbow	No	L	Intact	Yes	2
14	Intact	Yes	Absent below elbow	No	L	Intact	Yes	2
15	Absent below elbow	No	Intact	Yes	R	Intact	Yes	1
16	Intact	Yes	Absent below elbow	No	L	Intact		
17	Absent below elbow	No	Intact	Yes	R	Intact	Yes	1
18	Absent below elbow	No	Intact	Yes	R	Intact	Yes	3
19	Absent below wrist	No	Intact	Yes	R	Intact	No	-
20	Absent below elbow	No	Intact	Yes	R	Intact	Yes	1
21	Intact	Yes	Absent below elbow	No	L	Intact	No	-
22	Intact	Yes	Absent below elbow	No	L	Intact	Yes	1
23	Intact	Yes	Absent below wrist	No	L	Intact	Yes	2
24	Partial hand, 1 digit	No	Intact	Yes	R	Intact	Yes	5
25	Intact	Yes	Absent above elbow	No	L	Intact	Yes	5
26	Absent below elbow	No	Absent below shoulder	No	L	Feet	No	-
27	Intact	Yes	5 small digits	Yes	L	Residual	No	-
28	Intact	Yes	Short thumb, 1 non-jointed digit	Yes	L	Intact	No	-
29	Partial hand, 2 digits	Yes	Intact	Yes	R	Both		
30	Absent below elbow	No	Intact	Yes	R	Intact	No	-
31	Intact	Yes	Absent below wrist	No	L	Intact	Yes	5
32	Intact	Yes	Absent below wrist	No	L	Intact	No	-
33	Absent below elbow	No	Intact	Yes	R	Intact	Yes	2

Table S2

Details of adults with a limb difference. Pincer: defined as the ability to grasp a small object between the thumb and index finger; Dominant limb: R=right, L=left. Used limb: defined as use of intact/residual/both upper limbs, or feet. For prosthesis usage, 1 indicates daily usage > 8 hours per day, 2 indicates daily usage 4-8 hours per day, 3 indicates daily less than 4 hours per day, 5 indicates rare usage.

ID	Limb Difference				Dominant Limb	Used Limb	Prosthesis	
	Left	Pincer	Right	Pincer			Fitted	Use
1	Partial hand, 1 digit	No	Partial hand, 1 digit	No	R	Residual	No	-
2	5 digits, limited function	Limited	Intact	Yes	R	Intact	No	-
3	Partial hand, 1 digit	No	Partial hand, 3 digits	Yes	R	Residual	No	-
4	Absent below elbow	No	Intact	Yes	R	Intact	Yes	5
5	Absent below elbow	No	Intact	Yes	R	Intact	Yes	6
6	Intact	Yes	Partial hand, 2 digits	Yes	L	Both	No	-
7	Partial hand, 3 digits	No	Partial hand, 4 digits	No	R	Residual	No	-
8	Partial hand, no digits	No	Intact	Yes	R	Intact	Yes	2
9	Intact	Yes	Absent below elbow	No	L	Intact	Yes	-
10	Absent below elbow	No	Intact	Yes	R	Intact	No	-
11	Absent below elbow	No	Intact	Yes	R	Intact	Yes	3
12	Intact	Yes	Partial hand, no digits	No	L	Intact	No	-
13	Partial hand, 3 digits	Limited	Partial hand, 3 digits	Yes	R	Residual	No	-
14	5 digits, non-functional	No	Intact	Yes	R	Intact	No	-
15	Intact	Yes	Partial wrist, no digits	No	L	Intact	Yes	-
16	Intact	Yes	Partial hand, 2 digits	Yes	L	Residual	No	-
17	4 digits, non-functional	No	4 digits, non-functional	No	R	Intact	No	-
18	Absent below elbow	No	Intact	Yes	R	Intact	Yes	5
19	Intact	Yes	Absent below elbow	No	L	Intact	No	-
20	Absent above elbow	No	Absent above elbow	No	L	Feet	No	-
21	Intact	Yes	Absent above elbow	No	L	Intact	No	-
22	Absent below wrist	No	Intact	Yes	R	Intact	Yes	2
23	Absent below elbow	No	Intact	Yes	R	Intact	Yes	3
24	Intact	Yes	Partial hand, 4 digits	Yes	L	Both	No	-
25	Partial hand, 2 digits	No	Intact	Yes	R	Intact	No	-

Table S3

Details of children with a limb difference. Pincer: defined as the ability to grasp a small object between the thumb and index finger. Dominant limb: R=right, L=left. Used limb: defined as use of intact/residual/both upper limbs, or feet. Prosthesis use is measured as 1: use everyday, 2: use 5 days a week, 3: use weekly, 4: use monthly, 5: use a few times a year.

Trial Name	Adult: LD vs NLD	Children: LD vs NLD	NLD: Adult vs Children	LD: Adult vs Children
Balance	52% [47%, 56%]	50% [45%, 55%]	50% [45%, 55%]	47% [41%, 53%]
BalanceUnder	58% [53%, 62%]	51% [46%, 56%]	50% [45%, 54%]	60% [54%, 66%]
Basic	53% [48%, 57%]	50% [45%, 55%]	56% [51%, 60%]	53% [47%, 59%]
Bridge	55% [50%, 60%]	50% [46%, 55%]	50% [46%, 55%]	54% [48%, 59%]
Catapult	47% [43%, 52%]	53% [48%, 58%]	51% [47%, 56%]	56% [51%, 62%]
FallAlt	48% [43%, 52%]	58% [53%, 63%]	57% [52%, 61%]	61% [55%, 67%]
Falling_A	51% [46%, 56%]	60% [55%, 65%]	65% [60%, 70%]	55% [50%, 61%]
Gap	47% [43%, 52%]	51% [46%, 56%]	47% [42%, 51%]	48% [42%, 54%]
GoalMove	53% [48%, 58%]	47% [42%, 52%]	54% [49%, 59%]	47% [42%, 53%]
Remove	52% [48%, 57%]	51% [46%, 56%]	62% [57%, 66%]	51% [45%, 57%]
SeeSaw	54% [50%, 59%]	51% [46%, 56%]	52% [47%, 56%]	47% [41%, 53%]
TableCreative	47% [42%, 51%]	50% [45%, 55%]	56% [51%, 60%]	62% [56%, 68%]
Trap	52% [48%, 57%]	56% [51%, 61%]	54% [50%, 59%]	54% [49%, 60%]
Unbox	50% [46%, 55%]	53% [48%, 58%]	58% [53%, 63%]	50% [44%, 56%]

Table S4

Classification of first attempts between different groups. Numbers in brackets represent bootstrapped 95% confidence intervals on classification percentages.
